# Refractive Two-View Reconstruction for Underwater 3D Vision

**DOI:** 10.1007/s11263-019-01218-9

**Published:** 2019-11-18

**Authors:** François Chadebecq, Francisco Vasconcelos, René Lacher, Efthymios Maneas, Adrien Desjardins, Sébastien Ourselin, Tom Vercauteren, Danail Stoyanov

**Affiliations:** 1 Wellcome/EPSRC Centre for Interventional and Surgical Sciences (WEISS), London, UK; 2 School of Biomedical Engineering & Imaging Sciences, King’s College London, London, UK

**Keywords:** Underwater imaging, Two-view Refractive Structure-from-Motion, Flat refractive geometry

## Abstract

Recovering 3D geometry from cameras in underwater applications involves the Refractive Structure-from-Motion problem where the non-linear distortion of light induced by a change of medium density invalidates the single viewpoint assumption. The pinhole-plus-distortion camera projection model suffers from a systematic geometric bias since refractive distortion depends on object distance. This leads to inaccurate camera pose and 3D shape estimation. To account for refraction, it is possible to use the axial camera model or to explicitly consider one or multiple parallel refractive interfaces whose orientations and positions with respect to the camera can be calibrated. Although it has been demonstrated that the refractive camera model is well-suited for underwater imaging, Refractive Structure-from-Motion remains particularly difficult to use in practice when considering the seldom studied case of a camera with a flat refractive interface. Our method applies to the case of underwater imaging systems whose entrance lens is in direct contact with the external medium. By adopting the refractive camera model, we provide a succinct derivation and expression for the refractive fundamental matrix and use this as the basis for a novel two-view reconstruction method for underwater imaging. For validation we use synthetic data to show the numerical properties of our method and we provide results on real data to demonstrate its practical application within laboratory settings and for medical applications in fluid-immersed endoscopy. We demonstrate our approach outperforms classic two-view Structure-from-Motion method relying on the pinhole-plus-distortion camera model.

## Introduction

**Fig. 1:**
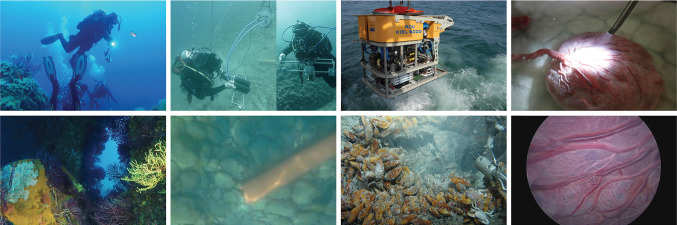
Example of submarine and fluid-immersed computer vision applications. From the left column to the right: submarine exploration using a camera embedded in a waterproof casing (images courtesy of Mr. Giorgio Capelli, FiasParma, Federazione Italiana Attività Subacquee Parma, national park of Port-Cros and Porquerolles), underwater object recognition for the development of Autonomous Underwater Vehicles[images courtesy of Robotics and Intelligent Machines Laboratory, University of Parma, Marine Autonomous Robotics for InterventionS project (Lodi Rizzini et al. 2015; Oleari et al. 2015)], Remotely Operated Vehicle KIEL 6000 used for submarine exploration (images courtesy of GEOMAR Helmholtz Centre for Ocean Research Kiel), laboratory experiment reproducing fetoscopic examination of a human placenta

Underwater imaging and 3D reconstruction are important for a variety of practical applications ranging from submarine exploration and robot guidance to fluid-immersed endoscopic surgical procedures such as arthroscopy or fetoscopy (see examples shown in Fig. 1). The problem of recovering both the 3D geometry of the environment and the camera motion when operating under water requires adaptation of the normal pinhole-plus-distortion camera model because non-linear refractive distortion occurs at the interface between the liquid and the camera’s housing. This means that the multi-view geometry of the problem is also influenced and additional considerations are required in order to formulate reconstruction as a Refractive Structure-from-Motion (RSfM).

**Fig. 2:**
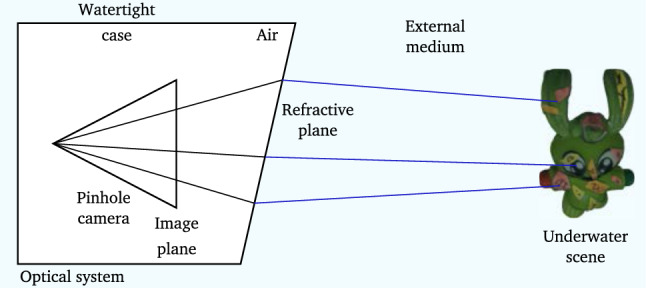
Refractive camera model. We assume the underwater imaging system can be modelled by a pinhole camera embedded within a watertight case. The underwater scene is observed through a flat and thin refractive interface

**Fig. 3:**
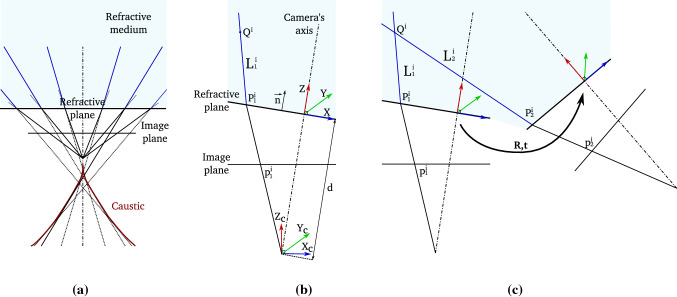
**a** Illustration of the ray-based camera model, where the rays refracted at the refractive interface of the optical system form a caustic. Due to this refraction, the single viewpoint assumption is not valid. The axial camera model can be defined by the envelope tangent to the refracted rays. As such, the caustic is a measure of the deviation from the pinhole camera model. **b** Illustration of the refractive camera model assuming a single, thin refractive interface attached to the camera. We assumed the refractive plane position and orientation to be known. **c** Two-view refractive geometry assuming the refractive model from (**b**)

When a camera is immersed in a fluid, light rays undergo refraction while passing through media with different optical density. The refractive distortion is defined by Snell’s law and depends on the refractive index of traversed medium as well as the incidence angle of the incoming light ray (Hecht 1998). Dome windows can be used to efficiently compensate for refractive distortion. They however exhibit severe field curvature, they are unwieldy and need to be specially engineered (Luczyński et al. 2017). Flat-pane windows are more flexible and affordable but they do not compensate for refractive distortion (see Fig. 2). Without taking into account refraction, it can be demonstrated that incoming light rays coming from the scene do not intersect anymore in a single center of projection and therefore the Single ViewPoint (SVP) assumption becomes invalid (Glaeser and Schröcker 2000; Treibitz et al. 2012, see Fig. 3a).

As a result, if a camera is calibrated in air assuming the pinhole model, calibration parameters cannot be used effectively once the camera is immersed within the liquid. While adapting the intrinsic camera parameters and distortion coefficients can compensate for refraction to some degree and for a limited working distance (Kang et al. 2012a; Treibitz et al. 2012), this approach introduces a systematic geometric bias which affects 3D measurements and camera pose estimation (Sedlazeck and Koch 2012). Indeed, refractive distortion directly depends on the incidence angle of the incoming light rays and as such on the scene depth [i.e. the 2D coordinates of a 3D point in the image plane directly depends on its depth (Treibitz et al. 2012)].

It has been demonstrated that image formation through a flat refractive interface can be modelled by an axial camera (Agrawal et al. 2012). However, such ray-based camera models are difficult to accurately calibrate and subsequently to use in practice due to the high dimensionality of their parametrisation (Glaeser and Schröcker 2000). The axial camera can also be regarded as a particular case of the generalised camera model for which multiple-view geometry has been extensively studied (Sturm et al. 2005; Sturm 2005; Li et al. 2008). However, this model is particularly sensitive to noise when considered for modelling monocular axial cameras whose image formation process can be efficiently approximated by a pinhole camera. Even if well calibrated, 3D reconstruction with a moving generalised camera remains even more challenging under water due to image quality degradations in such context. Approaches to solve this problem typically make prior assumptions, such as knowledge of the camera orientation (Chang and Chen 2011), or considering that the camera moves behind a fixed refractive interface (Chari and Sturm 2009) which is not suitable to estimate the motion of an immersed camera embedded within a waterproof housing.

An alternative approach to using generalised camera models is to explicitly consider one or more interfaces separating the optical system from the external medium. The media on either side of the interfaces have different refractive indexes causing refraction (Agrawal et al. 2012). By explicitly modelling this configuration’s geometry, as shown in Fig. 3b, it is possible to form a refractive camera model with parameters that account for refractive distortion. Algorithms for 3D reconstruction can then rely on the refractive geometric constraints (Jordt-Sedlazeck et al. 2013; Jordt-Sedlazeck and Koch 2013; Jordt et al. 2016). Using refractive geometry, the two-view relative pose problem can be solved iteratively followed by bundle adjustment by associating each 2D image point to a virtual perspective camera (Agrawal et al. 2012). This formulation avoids directly computing the refractive re-projection error, which is computationally expensive as it requires solving a 12th degree polynomial (Treibitz et al. 2012). Dense 3D reconstruction can then be obtained by using a refractive plane sweep algorithm (Jordt-Sedlazeck et al. 2013).

More recently, Luczyński et al. (2017) proposed the Pinax camera model which combines a virtual pinhole camera model with the axial camera model. It more particularly allows the pre-computation of a lookup-table for fast and accurate refraction correction. The Pinax model has furthermore demonstrated a high efficiency for stereo 3D reconstruction under water.

A notable current limitation of existing methods for estimating relative camera motion under water is that they are highly sensitive to noise and this results in a major practical limitation given that underwater images are subject to complex light scattering as well as medium turbidity, which makes precise point matches difficult to establish. In certain application domains, for example fluid-immersed endoscopic surgical procedures, accurate correspondence can be extremely challenging due to paucity of reliable features, medium properties and the restricted workspace of the moving camera. This means that stable and robust methods for estimating the refractive two view geometry and relative pose of cameras are an important area for further development in underwater vision and imaging.

### Contribution

We propose a novel stereo 3D-reconstruction method for the case of a pinhole camera looking through a thin refractive interface, where the position and orientation of this interface are known (see Fig. 2). Our approach is particularly flexible and can be applied to various applications of underwater imaging and vision behind a thin refractive interface. This paper extends our previous work (Chadebecq et al. 2017) by:

i. providing with an explicit formulation of the refractive Fundamental relationship (rF), further developing the previous theoretical derivation of the two-view geometry for a fixed refractive plane (Chari and Sturm 2009) (see Sect. 4). By appropriately formulating the rF, we propose a robust stereo reconstruction method able to withstand complex underwater imaging conditions,
ii. extensively evaluating our approach for different underwater scenarios ranging from fluid-immersed medical endoscopy to deep underwater imaging. To demonstrate our contribution to the refractive geometry of underwater vision, we evaluate the effectiveness and improvements on numerical stability of our approach on synthetic and real data over classic two-view Structure-from-Motion (2View SfM) relying on the pinhole-plus-distortion perspective camera model. To highlight a particular application for two-view Refractive Structure-from-Motion (2View RSfM), we show results for fetoscopy, where an endoscope is used to guide vessel photo-coagulation on the placental surface within the amniotic fluid.

### Appendices

For the sake of clarity, the refractive forward projection equation introduced in Agrawal et al. (2012) is recalled in appendix 1 and the refractive single-view geometry introduced in Chari and Sturm (2009) is recalled in “Appendix B”.

## Underwater Imaging Model and Refractive Structure-from-Motion: Related Work

To provide the necessary background to our work, we first introduce the camera models for refractive imaging which are generally considered for submarine and underwater exploration. A detailed review of these models can be found in Sedlazeck and Koch (2012). We then describe state of the art RSfM approaches. An exhaustive survey of these approaches can be found in Jordt (2014). A complementary survey on underwater 3D reconstruction can be found in Massot-Campos and Oliver-Codina (2015).

### Refractive Image Formation Model

In previous works, three different image formation models have been used for refractive imaging and vision: the pinhole model (Kang et al. 2012a), the ray-based and generalised model (Chari and Sturm 2009), and the refractive model (Agrawal et al. 2012; Jordt 2014).

*Adapted Pinhole Camera Model* Most underwater computer vision literature has previously relied on the classic pinhole camera model of perspective projection. This choice is mainly motivated by its simplicity, general popularity and efficiency despite the known systematic geometric bias due to refraction (Glaeser and Schröcker 2000; Treibitz et al. 2012). Several papers have demonstrated that focal length and distortion parameters could be adapted to partially compensate for refraction effects (Fryer and Fraser 1986; Lavest et al. 2000; Kang et al. 2012a). Such assumption remains valid assuming the refractive plane is fronto parallel to the image plane of the camera. If it is tilted, symmetric or irregular distortion functions should be considered (You et al. 2016; Agrawal and Ramalingam 2013). Moreover, the adapted pinhole camera model is valid within a limited range of distance (the range of distances considered for camera calibration) as refractive distortion depends on the depth of the scene (Treibitz et al. 2012). The underlying geometry of rays actually defines an axial camera model (Agrawal et al. 2012). The influence of scene depth on the approximation error of the adapted pinhole model has been evaluated in Luczyński et al. (2017). They simulated an underwater scene observed at a reference distance of 2 m corresponding to the distance at which the camera was synthetically calibrated. They reported an approximation error of the order of 20 pixels for a 3D point located 50 cm away from the reference distance. This error corresponded to the average 2D distance between a set of 3D points projected within the image plane using the adapted pinhole and refractive camera models.

*Ray-Based and Generalised Camera Model* A complex but comprehensive approach is to associate each image pixel within the image to a light ray, similarly to the raxel model (Grossberg and Nayar 2005), and this has been used effectively in underwater imaging (Glaeser and Schröcker 2000; Sturm and Ramalingam 2004; Sturm et al. 2005; Treibitz et al. 2012). The imaging system can be characterized by its caustic, which is defined as the surface tangential to the bundle of rays intersecting the camera axis (see the illustration in Fig. 3a). In practice, this model is difficult to calibrate due to its large number of parameters (Sturm and Ramalingam 2004). An alternative is the generalised camera model which describes a generic and unified image formation process for both single and non-single viewpoint cameras (Grossberg and Nayar 2001). Calibration of the generalised model and its use in multiple-view geometry have been studied (Sturm et al. 2005) and the approach has also been extended to underwater imaging assuming a camera moving behind a fixed refractive plane (Chari and Sturm 2009).

*Refractive Camera Model* Explicitly considering refraction at one or multiple flat-refractive planes (sharing the same orientation) has been proposed in a range of works (Agrawal et al. 2012; Jordt-Sedlazeck and Koch 2012; Yau et al. 2013; Chen and Yang 2014). We adopt this approach considering a single and thin refractive interface (illustrated in Fig. 3b). Such configuration is particularly adapted to underwater camera for which entrance lens is in direct contact with the external medium. This is for instance the case of fluid-immersed endoscopic imaging system used for medical procedure such as fetoscopy or arthroscopy. Moreover, it has been shown in Agrawal et al. (2012) that such relatively simple model (compared to the thick interface case) is capable of approximating both single and multiple-layer systems which allows us to propose a generic method for 2View RSfM.

*Combined Camera Model* The Pinax model proposed by Luczyński et al. (2017) combines a virtual pinhole camera model with the axial camera model. It assumes the distance between the refractive plane and the camera’s optical center is close to the optimal distance for which the interval defined by the intersection of the light rays and the camera’s axis is minimal. Under this assumption, it can be shown that the pinhole model efficiently approximates the underwater image formation process. As such, each image point of the virtual pinhole camera can be back-projected onto a virtual pinax plane (virtual plane perpendicular to the camera’s axis and situated at a reference distance in the scene) and then projected forward onto the physical camera (modelled by an axial camera). This allows the pre-computation of a lookup-table for fast and accurate refraction correction. Moreover, it is also particularly convenient as it only requires an in-air calibration of the underwater camera system. It is however sensitive to external medium refractive index changes and the distance of the scene can influence 3D reconstruction accuracy as it directly relies on the SVP approximation (which is an important limitation considering applications such as fluid-immersed endoscopy).

### Refractive 3D Reconstruction

The problem of recovering 3D geometry in the presence of refraction has been studied in the past and there is a recent survey on active and passive 3D reconstruction methods for underwater imaging (Massot-Campos and Oliver-Codina 2015). In this section, we briefly review the most relevant approaches to this paper. The majority of approaches rely on standard SfM methods assuming the pinhole-plus-distortion camera model can compensate for refraction, which is known as the SVP assumption (Yu et al. 2003). It has been demonstrated that adapting radial lens distortion and focal length can compensate for refraction to a certain extent (Lavest et al. 2000) but this only leads to acceptable pose and 3D shape estimation within a limited range of distance of the scene (Kang et al. 2012b). There still exists a systematic geometric bias and estimated camera poses absorb some of the errors due to modelling (Sedlazeck and Koch 2012). Moreover, Jordt-Sedlazeck and Koch (2013) showed that measurement errors are particularly significant when the refractive interface is not strictly fronto-parallel to the image plane. As such, standard SfM methods remain poorly reliable under water due to drifting of camera poses estimations.

Few dedicated RSfM methods relying on ray-based models have been proposed. This is notably due to the difficulty of accurately calibrating such a system and its high sensitivity to noise. It has moreover been demonstrated that physical-based models outperform the generalised camera model (see Mouragnon et al. (2009) for SfM in the context of deep underwater imaging). Two theoretical works remain however particularly relevant to the refractive geometry of underwater vision. Considering the case of a camera moving behind a fixed refractive plane such as a camera seeing through an aquarium (fixed interface scenario), Chari and Sturm (2009) derived the rF relationship. It is however defined by a $12\times 12$ matrix whose estimation is computationally unstable. An alternative ray-based model has been proposed in Chaudhury et al. (2015). It allows to express the underlying refractive geometry as a direct extension of the projective geometry. The authors assume refraction happens at the camera centre only allowing them to geometrically estimate the relationship between the observed image point and the one obtained if refraction had not taken place [following a similar idea to the division model for distortion (Fitzgibbon 2001)]. By correcting image coordinates, the fundamental matrix can be directly estimated, while the external camera parameters and the 3D reconstruction are obtained up to a similarity.

Most of the RSfM approaches rely on a physical-based model by explicitly considering a pinhole camera looking through a refractive plane. The case of a fixed interface is considered in Chang and Chen (2011). They assume the camera embeds an Inertial Measurement Unit (IMU) and therefore both pitch and yaw of the camera are known. They proposed a closed-form solution to the absolute pose estimation, however it requires to know the vertical displacement of the camera. The case of two cameras embedded in different watertight casing is considered in Kang et al. (2012b). They propose an optimal solution to the relative translation problem under $\mathrm {L}_\infty$ assuming camera rotation is known. This solution is then extended to the unknown camera rotation case assuming a thin refractive plane parallel to both image plane of the cameras. More recently, Haner and Aström (2015) developed efficient minimal solvers for the cases considered in Chang and Chen (2011), Kang et al. (2012b) to solve for the absolute pose problem (camera observing known structure through a known refractive plane). For the general case of deep underwater imaging, Jordt et al. (2016) developed a complete RSfM framework by combining their previous works (Jordt-Sedlazeck and Koch 2013; Jordt-Sedlazeck et al. 2013). Relative camera motion between two successive views is estimated by relying on the flat refraction and coplanarity constraints derived from Agrawal et al. (2012) [respectively equations (12),  (13)]. They then proposed a non-linear refractive bundle adjustment by extending (Ramalingam et al. 2006). It is based on the idea that a virtual camera can be associated to each 2D point into which the corresponding 3D point can be projected perspectively. Finally, dense depth estimation is obtained by using a refractive plane sweep algorithm (Gallup et al. 2007). This method requires a good initialisation, more particularly for two-view pose estimation, and relies on a costly iterative algorithm.

It is worth to note that RSfM has also been considered to solve for the absolute scale ambiguity inherent to SfM (Shibata et al. 2015a, b). Indeed, knowing the position and orientation of the interfaces theoretically yields to the absolute camera motion as relative pose is no more invariant to scale change in camera translation (Jordt-Sedlazeck and Koch 2013). The authors of Shibata et al. (2015a) suggest to place a thick refractive plate in front of a pinhole camera in order to infer the absolute scale of a scene. The coplanarity constraint is extended to two views and directly solved using a least squares method. However, this approach is particularly sensitive to noise and requires at least 17 points correspondences. It has been experimentally observed in Jordt et al. (2016) that RSfM methods are too sensitive to noise to be able to infer the absolute scale of a scene. This is particularly the case considering a thin refractive interface as we noticed during our experiments.

## Notation

For consistency, we adopt the mathematical notation used in Chari and Sturm (2009) (see Fig. 3b). The world coordinate frame (*X*, *Y*, *Z*) is arbitrarily set up in the first view. The *Z*-axis lies on the camera’s axis defined as the line passing through the normal of the refractive interface (${\mathbf {n}}=(0\ 0\ 1)^\top$) and the camera optical centre. It is important to note that we here distinguish between the camera’s axis (i.e. the camera is composed of a pinhole camera looking through a refractive interface) and the pinhole camera’s axis $Z_c$. The camera’s and pinhole camera’s axis are thus not necessarily aligned. The *X* and *Y* axes lie on the refractive plane and respectively align with the orthographic projection of $X_c$-axis and $Y_c$-axis of the camera coordinate frame onto the refractive plane. The pose of the camera in the first view is expressed as $\mathsf {P}_1 = \mathsf {R}_r^{-1}(\mathsf {I} - \mathbf {t}_r)$ where $\mathsf {I}$ is the $3\times 3$ identity matrix, $\mathsf {R}_r$ is the $3\times 3$ rotation matrix corresponding to the refractive plane orientation relative to the camera coordinate frame and $\mathbf {t}_r = (0\ 0\ {d})^\top$ is the refractive plane position. The interface to camera centre distance along the camera’s axis is denoted by *d*. A 2D image point *i* observed in view *j* is denoted by $\mathbf {p}^{i}_j = (x\ y\ 1)^\top$. We denote as $\mathbf {P}^{i}_j = (x\ y\ z\ 1)^\top$ the 3D point of incidence (point lying on the refractive interface) related to the 3D point $\mathbf {Q}^{i}$ projected in $\mathbf {p}^{i}_j$.

Light rays are defined by a starting point (e.g. a point of incidence) and a direction vector. Direction vectors associated to incident light rays (travelling within water) are denoted by $\mathcal {L}^{i}_j$. Direction vectors associated to their corresponding refracted light rays (travelling within the water tightness housing) are denoted by $\mathcal {P}^{i}_j$. They are directly derived, in the world coordinate system, from the expression $\mathcal {P}^{i}_{j} = ({p_x}_{j}^{i}\ {p_y}_{j}^{i}\ {p_z}_{j}^{i})^\top = ( \mathsf {R}_r^{-1} \frac{-\mathsf {K}^{-1}\mathbf {p}^{i}_{j}}{\Vert \mathsf {K}^{-1}\mathbf {p}^{i}_{j}\Vert })^\top$ where $\mathsf {K}^{-1}$ corresponds to the internal camera parameters of the pinhole camera. The Plücker coordinates of incident light rays are denoted by $\mathbf {L}_{j}^{i}=({\mathbf {L}_0}_j^i,\dots ,{\mathbf {L}_6}_j^i)^\top$ (Yu et al. 2003). As such, ${\mathbf {L}_{(a,b,c)}}_j^i$ defines a vector composed of the elements ${\mathbf {L}_a}_j^i$, ${\mathbf {L}_b}_j^i$ and ${\mathbf {L}_c}_j^i$ of ${\mathbf {L}}_j^i$.

The lifted coordinates of the n-dimensional vector $\mathbf {v}$ can be obtained by vectorising the upper triangular part of the matrix $\mathbf {v}\mathbf {v}^\top$. Therefore, the expressions $\hat{\mathbf {v}}=({v_x}^2\ v_xv_y\ {v_y}^2\ v_xv_z\ v_yv_z{v_z}^2)^\top$ and ${\hat{\mathbf {v}}}~=~({v_u}^2\ v_uv_v\ {v_v}^2 v_uv_w\ v_vv_w\ {v_w}^2 v_uv_x\ v_vv_x\ v_wv_x{v_x}^2\ v_uv_y\ v_vv_y\ v_wv_y\ v_xv_y {v_y}^2\ v_uv_z\ v_vv_z\ v_wv_z\ v_xv_z\ v_yv_z\ {v_z}^2)^\top$ respectively define the lifted coordinates of the three-dimensional vector $\mathbf {v}=(v_x\ v_y\ v_z)^\top$ and the six-dimensional vector $\mathbf {v}=(v_u\ v_v\ v_w\ v_x\ v_y\ v_z)^\top$. If two vectors are related by a linear transformation $\mathsf {T}$ such as $\mathbf {v_2}~=~\mathsf {T} \mathbf {v_1}$, their lifted coordinates are related by the expression ${\hat{\mathbf {v}_2}}~=~\mathsf {D}^{-1}\mathsf {S}(\mathsf {T}~\otimes ~\mathsf {T})\mathsf {S}^\top {\hat{\mathbf {v}_1}}$ (Sturm and Barreto 2008). The symbol $\otimes$ refers to the Kronecker product. The design matrix *S* is a binary matrix of size $\frac{n^2+n}{2}\times n^2$. In the three-dimensional case, we denote *S* by $S_t$ which is defined as:

$$\begin{aligned} \mathsf {S_t} = \left( \begin{array}{ccccccccc} 1 &{} 0 &{} 0 &{} 0 &{} 0 &{} 0 &{} 0 &{} 0 &{} 0 \\ 0 &{} 1 &{} 0 &{} 1 &{} 0 &{} 0 &{} 0 &{} 0 &{} 0 \\ 0 &{} 0 &{} 0 &{} 0 &{} 1 &{} 0 &{} 0 &{} 0 &{} 0 \\ 0 &{} 0 &{} 1 &{} 0 &{} 0 &{} 0 &{} 1 &{} 0 &{} 0 \\ 0 &{} 0 &{} 0 &{} 0 &{} 0 &{} 1 &{} 0 &{} 1 &{} 0 \\ 0 &{} 0 &{} 0 &{} 0 &{} 0 &{} 0 &{} 0 &{} 0 &{} 1 \end{array} \right) \end{aligned}$$

In the six-dimensional case, we denote *S* by $S_s$. For the sake of brevity, we provide the matrix index corresponding to the non-null value (i.e. $\mathsf {S}_s{^i_j}$ corresponds to the entry in the *i*th row and *j*th column of the matrix).The matrix $\mathsf {D}$ is a diagonal matrix of size $m \times m$ where *m* is the number of rows of *S*. Its entries are given by the expression $\mathsf {D}_{ii} = \sum _{j=1}^{k}S_{ij}$ where *k* is the number of columns of *S*. Therefore, $\mathsf {D}_t = {{\,\mathrm{diag}\,}}(1\, 2\, 1\, 2\, 2\, 1)$ and $\mathsf {D}_s={{\,\mathrm{diag}\,}}(1\, 2\, 1\, 2\, 2\, 1\, 2\, 2\, 2\, 1\, 2\, 2\, 2\, 2\, 1\, 2\, 2\, 2\, 2\, 2\, 1)$. These relationships can easily be extended to *n* dimensions.

## Refractive Geometry

The refractive projection equation introduced in Chari and Sturm (2009) is recalled in “Appendix B”. We here derive the two-view refractive relationship for the practical case of an underwater camera whose watertight case window can be modelled by a thin refractive plane.

### Two-view Refractive Geometry

We here consider two views of an underwater scene. The incident light ray of direction-vector $\mathcal {L}_2^i$ stemming from the point $\mathbf {Q}^{i}$ is projected in $\mathbf {p}^{i}_2$ in the second view (see illustration Fig. 3c). The Plücker coordinates of $\mathcal {L}_2^i$ are given by:

1 $$\begin{aligned} \mathbf {L}_2^i = \mathsf {T}\ (\lambda {p_x}_{2}^{i}\ \ \lambda {p_y}_{2}^{i} \ \ {\gamma _z}^i_2\ \ {-d\frac{{p_y}_{2}^{i}}{{p_z}_{2}^{i}}{\gamma _z}^i_2} \ \ d\frac{{p_x}_{2}^{i}}{{p_z}_{2}^{i}}{\gamma _z}^i_2 \ \ 0)^\top \end{aligned}$$

where $$\mathsf {T} = \left( \begin{array}{cc} \mathsf {R} &{} \mathsf {0} \\ {[t]}_x\mathsf {R} &{} \mathsf {R} \end{array} \right) $$ and $${[t]}_x = \left( \begin{array}{ccc} 0 &{} -t_z &{} t_y \\ t_z &{} 0 &{} -t_x \\ -t_y &{} t_x &{} 0 \end{array} \right) $$ corresponds to the camera pose (Sturm and Barreto 2008). The scalar $\lambda =\frac{\mu _1}{\mu _2}$, where $\mu _1$ corresponds to the refractive index within the camera housing (generally equal to 1) and $\mu _2$ corresponds to the refractive index of the refractive medium. For brevity’s sake we define ${\gamma _z}^i_j~=~\sqrt{1-\lambda ^2+\lambda ^2 {{p_z}_{j}^{i}}^2}$.

By reordering the expression of $\mathbf {L}_2^i$, we can extract vector components ${\mathbf {L}_{(6,1,2)}}^i_2$ and ${\mathbf {L}_{(4,5,3)}}^i_2$:

2 $$\begin{aligned} \begin{pmatrix} {\mathbf {L}_{(6,1,2)}}^i_2 \\ {\mathbf {L}_{(4,5,3)}}^i_2 \end{pmatrix} = \mathsf {T'} \begin{pmatrix} \lambda t_u &{} 0 \\ 0 &{} t_v \end{pmatrix} \begin{pmatrix} {\mathcal {P}}^{i}_{2} \\ {\gamma _z}^i_2 \frac{{\mathcal {P}}^{i}_{2}}{{{p_z}_{2}^{i}}^2}\end{pmatrix} \end{aligned}$$

where $$\mathsf {t}_u = \left( \begin{array}{ccc} 0 &{} 0 &{} 0 \\ 1 &{} 0 &{} 0 \\ 0 &{} 1 &{} 0 \end{array} \right) $$ and $$ \mathsf {t}_v = \left( \begin{array}{ccc} 0 &{} {-d} &{} 0 \\ d &{} 0 &{} 0 \\ 0 &{} 0 &{} 1 \end{array} \right) $$. The matrix $\mathsf {T'}$ corresponds to the camera pose matrix $\mathbf{T}$ reorganised:

3 $$\begin{aligned} \mathsf {T'} = \left( \begin{array}{cccccc} R_{33} &{} {[t]_{x}}_{3:}R_{.1} &{} {[t]_{x}}_{3:}R_{.2} &{} R_{31} &{} R_{32} &{} {[t]_{x}}_{3:}R_{.3} \\ 0 &{} R_{11} &{} R_{12} &{} 0 &{} 0 &{} R_{13} \\ 0 &{} R_{21} &{} R_{22} &{} 0 &{} 0 &{} R_{23} \\ R_{13} &{} {[t]_{x}}_{1:}R_{:1} &{} {[t]_{x}}_{1:}R_{:2} &{} R_{11} &{} R_{12} &{} {[t]_{x}}_{1:}R_{:3} \\ R_{23} &{} {[t]_{x}}_{2:}R_{:1} &{} {[t]_{x}}_{2:}R_{:2} &{} R_{21} &{} R_{22} &{} {[t]_{x}}_{2:}R_{:3} \\ 0 &{} R_{31} &{} R_{32} &{} 0 &{} 0 &{} 0 \end{array} \right) \end{aligned}$$

The vector $R_{:i}$ identifies the *i*th column of the camera rotation matrix and we define $R_{.i} = [R_{1i}\ \ R_{2i}\ \ 0]^\top$. Similarly, ${[t]_{x}}_{i:}$ identifies the *i*th row of the matrix ${[t]_{x}}$.

Expressing concisely the lifted coordinates of the aforementioned vector components is not obvious and eliminating the square root in ${\gamma _z}{^i_2}$ leads to a complex expression. We can nevertheless extract the lifted vector components $\left( {\widehat{\mathbf {L}}}{^\top _{(6,1,2)}}{^i_2} \ \ {\widehat{\mathbf {L}}}{^\top _{(4,5,3)}}{^i_2} \right)$ from the expression of $\widehat{ \left( {\mathbf {L}}{^\top _{(6,1,2)}}{^i_2} \ \ {\mathbf {L}}{^\top _{(4,5,3)}}{^i_2} \right) }$ obtained by vectorizing the upper triangular part of the matrix ${\left( {\mathbf {L}}{^\top _{(6,1,2)}}{^i_2} \ \ {\mathbf {L}}{^\top _{(4,5,3)}}{^i_2}\right) } {\left( {\mathbf {L}}{^\top _{(6,1,2)}}{^i_2} \ \ {\mathbf {L}}{^\top _{(4,5,3)}}{^i_2}\right) }^\top$ (see Sect. 3). We can then derive the following relationship:

4 $$\begin{aligned} \left( {\widehat{\mathbf {L}}}{^\top _{(6,1,2)}}{^i_2} \ \ {\widehat{\mathbf {L}}}{^\top _{(4,5,3)}}{^i_2} \right) = \mathsf {N} \ {\widehat{\mathsf {T'}}} \ {\widehat{\mathsf {C}}} \ {\widehat{\left( {{\mathcal {P}}^{i}_{2}}^\top \ \frac{{{\mathcal {P}}^{i}_{2}}}{{{p_z}_{2}^{i}}^2}^\top \right) }} \end{aligned}$$

where $\mathsf {N}$ is a sparse change of basis binary matrix used to rearrange rows and reduce matrix dimensionality. It is defined by:

5 $$\begin{aligned} \mathsf {N}(^1_1,^2_2,^3_3,^4_4,^5_5,^6_6,^{\ 7}_{10},^{\ 8}_{14},^{\ 9}_{15},^{10}_{19},^{11}_{20},^{12}_{21}) = 1 \end{aligned}$$

The lifted coordinates of the camera pose matrix $\mathsf {T'}$ are given by ${{\widehat{\mathsf {T'}}}} = \mathsf {D}_s^{-1} \mathsf {S}_s \mathsf {T'}\otimes \mathsf {T'} \mathsf {S}_s^\top$. Matrix $\mathsf {D}_s$ and $\mathsf {S}_s$ are defined in Sect. 3.

The matrix *C*, which notably embeds the refractive parameters (interface position and medium refractive index), is defined by:

6 $$\begin{aligned} \mathsf {C} = \left( \begin{array}{cc} \lambda \mathsf {t}_u &{} 0 \\ 0 &{} {\gamma _z}^i_2 \mathsf {t}_v \end{array} \right) \end{aligned}$$

The lifted matrix ${\widehat{{\mathsf {C}}}}$ is derived using ${\widehat{{\mathsf {C}}}} = \mathsf {D}_s^{-1} \mathsf {S}_s \mathsf {C} \otimes \mathsf {C} \mathsf {S}_s^\top$.

Combining equations (22) and (4) allows us to define the refractive fundamental relationship:

7 $$\begin{aligned} \widehat{\left( {\mathcal {P}}^{i}_{2} \ \ \frac{{\mathcal {P}}^{i}_{2}}{{{p_z}_{2}^{i}}}\right) }^\top \mathsf {rF} \ \begin{pmatrix} \frac{\widehat{\mathcal {P}}_{1}^{i}}{{{p_z}_{1}^{i}}^2} \\ {\widehat{\mathcal {P}}_{1}^{i}} \end{pmatrix} = 0 \end{aligned}$$

The matrix $\mathsf {rF}$ is defined as:

8 $$\begin{aligned} \mathsf {rF} = {{\widehat{{\mathsf {C}}}}}^\top {{\widehat{\mathsf {T'}}}}^\top \mathsf {N}^\top \mathsf {M} \mathsf {D_s} \end{aligned}$$

where the matrix $\mathsf {M}$ is defined by:

9 $$\begin{aligned} \mathsf {M} = \left( \begin{array}{cc} (1-\lambda ^2) \mathsf {D}_t^{-1}\mathsf {S}_t\mathsf {t}_b^\top \otimes \mathsf {t}_b^\top \mathsf {S}_t^\top &{} 0 \\ \\ \lambda ^2 \mathsf {D}_t^{-1}\mathsf {S}_t\mathsf {t}_b^\top \otimes \mathsf {t}_b^\top \mathsf {S}_t^\top &{} -\lambda ^2 \mathsf {D}_t^{-1}\mathsf {S}_t\mathsf {t}_a^\top \otimes \mathsf {t}_a^\top \mathsf {S}_t^\top \end{array} \right) \end{aligned}$$

The matrix $$t_a=\left( \begin{array}{ccc} 1 &{} 0 &{} 0 \\ 0 &{} 1 &{} 0 \\ 0 &{} 0 &{} 0 \end{array} \right) $$ and $$t_b=\left( \begin{array}{ccc} 0 &{} 0 &{} 1 \\ 0 &{} -d &{} 0 \\ d &{} 0 &{} 0 \end{array} \right) $$. The complete derivation of the matrix M is recalled in “Appendix B” (Chari and Sturm 2009).

The rF matrix is of size $12\times 21$. It directly depends on refractive interface parameters (distance and orientation), as well as, the camera’s motion.

## Refractive Camera Pose Estimation

Directly estimating rF matrix entries from two-view correspondences is computationally unstable and practically difficult because it requires matching $12\times 21 = 252$ points across the image pair. The high degree of freedom of the rF matrix prevents closed-form algebraic solutions to provide with reliable estimates of its entries. These solutions are particularly sensitive to noise, as it can also be observed for the estimation of the generalised essential matrix considering a monocular axial camera. Even using a robust method such as RANdom Sample Consensus (RANSAC), we were not able to reliably estimate the rF matrix and as such extract absolute or relative cameras’ poses.

This suggested to rely on a two step relative pose estimation method. The relative camera motion is first estimated using the classic two-view SfM pipeline. Assuming the refractive geometry of the camera is calibrated (using Agrawal and Ramalingam 2013), its pose can then be refined using both the rF relationship and reprojection constraints. The rF matrix is computed thanks to equation (8). The reprojection constraint is based on the refractive forward projection equation (see “Appendix A”). It is worth to note that the rF constraint is also essential to remove outlier correspondences. This is a particularly sensitive key point in underwater imaging where images are highly affected by medium properties.

### Camera Calibration

We assume the camera to be calibrated both in air and under water. In air, calibration has been achieved using (Bouguet 2008). Underwater calibration consists in calibrating the camera considering both the single viewpoint approximation and the refractive camera model. The underwater pinhole calibration is similar to the calibration in air. Calibration of the position and orientation of the thin refractive plane has been performed using (Agrawal et al. 2012) relying on the same set of images. Whereas this method only requires a single view of the calibration target, we relied on the full set of underwater calibration images (20 images). We experimentally noticed a stable estimation of the interface orientation while the estimation of the interface depth suffered from a significant variance. We used the average interface depth estimated over the 20 underwater calibration images.

It is worth to note that calibrations have been performed under water before each experiment. As it will be showed using synthetic datasets in Sect. 6.1, slight changes in medium refractive index do not significantly impact camera pose estimation.

### Underwater Relative Pose Estimation

**Fig. 4:**
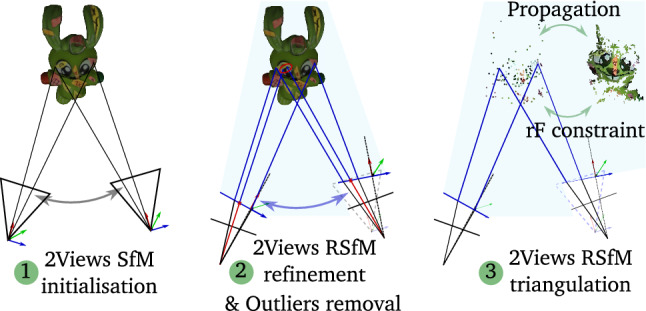
Overview of the proposed relative pose estimation method. The relative pose is first estimated using the classic fundamental constraint assuming the camera has been calibrated under water. The relative camera pose is then refined using the refractive Fundamental (rF) and reprojection constraints. Outlier Correspondences are discarded based on the rF constraint. Propagating key correspondences using (Stoyanov et al. 2010) and removing geometrically inconsistent correspondences using the rF constraint allowed us to obtain quasi dense reconstruction

The proposed relative pose estimation method is depicted in Fig. 4.

Our method is composed of the following steps. The first step relies on the single viewpoint assumption. After undistorting images using distortion parameters estimated under water, SIFT features are extracted and matched between each pair of views (Lowe 1999; Vedaldi and Fulkerson 2008; Daga et al. 2016). Along this step, fundamental matrix and relative camera motion are estimated using standard direct estimation approaches (Yu et al. 2003). The second step consists in refining the single viewpoint approximation. This is done by minimising the following non-linear least squares constraint based on the rF and reprojection constraints:

10 $$\begin{aligned} {\theta ^*},\mathbf {t^*} = \underset{\theta ,\mathbf {t}}{{\text {arg}}\,{\text {min}}}\; \sum _{i=1}^{n} FC_i(\theta ,\mathbf {t}) + \sum _{i=1}^{n} Proj_i(\theta ,\mathbf {t}) \end{aligned}$$

where the Euler angles $\theta =(\theta _x,\theta _y,\theta _z)^\top$ and $\mathbf {t}$ define the rotation and translation of the camera respectively. The indices $i=\{1,\dots n\}$ correspond to the point number. $FC_i$ corresponds to the refractive fundamental constraint defined in equation (7) and computed for the point *i*. The reprojection constraint $Proj_i$, computed for the point *i*, is defined by:

11 $$\begin{aligned} \begin{aligned} Proj_i(\theta ,\mathbf {t}) = \Vert \mathcal {P}_p \mathcal {P}_r (\theta _0,\mathbf {t}_0,\mathbf {Q}^i)&- \mathbf {q}^i_1 \Vert _2^2 + \\&\left\Vert \mathcal {P}_p \mathcal {P}_r(\theta ,\mathbf {t},\mathbf {Q}^i) - \mathbf {q}^i_2\right\Vert _2^2 \end{aligned} \end{aligned}$$

where $\mathbf {q}^i_1$ and $\mathbf {q}^i_2$ respectively correspond to the observations of the 3D point $\mathbf {Q}^i$ in the first and second view (i.e. corresponding to matching *i*). The refractive projection funtion $\mathcal {P}_r$ projects the 3D point $\mathbf {Q}^i$ onto the refractive plane.The refractive forward projection function is derived in Agrawal et al. (2012). Its definition is recalled in appendix 1, equation (21). We denote as $(\theta _0,\mathbf {t}_0)$ the camera’s pose corresponding to the first view. Without any loss of generality, we fixed $\theta _0=\mathbf {t}_0=(0,0,0)^\top$. Points lying on the refrative plane are projected onto the image plane thanks to the perspective projection matrix $\mathcal {P}_p$ (obtained by calibration).

We used a similar weight for the reprojection and the refractive fundamental constraints involved in cost function (10) as we experimentally observed that it allowed us to obtain the most accurate pose and 3D reconstruction estimates. As demonstrated by our experiments, the 2View SfM pose estimates computed in the first step of our method provided us with an accurate and reliable initialisation of camera poses sufficiently close to the optimum estimates. The minimisation problem is then solved using the Levenberg-Marquardt method (Marquardt 1963) and the rF matrix is used to reject outliers. We practically used a threshold of 0.1 pixels to truncate matches that do not conform to the refractive geometry.

We finally obtain quasi-dense stereo correspondences using an iterative growing scheme (Stoyanov et al. 2010). Again, outliers are discarded using the refractive geometry which leads to a final quasi-dense reconstruction.

### Discussion

The proposed 2View RSfM approach explicitly relies on refractive geometry. Unlike 2View SfM, relying on the adapted pinhole model, it does not suffer from a systematic geometric bias. Additionally, it allows us to efficiently discard mismatches which is of particular significance for application such as fluid-immersed endoscopy for which underwater artefacts generally prevent the reliable tracking of features (complex light scattering, medium turbidity, motion blur due to the manipulation of a flexible endoscope within a closed environment). By combining our approach with the method proposed in Stoyanov et al. (2010), we obtain quasi-dense reconstructions.

The proposed method nevertheless suffers from limitations. Because its second step relies on a costly non-linear minimisation (equation (10)), it is not real time. Furthermore, as its first step relies on 2View SfM it inherits from its limitations. Most particularly, it is sensitive to the same critical camera motions and it suffers of scale ambiguity (Yu et al. 2003). Similarly, the 3D densification step inherits from Stoyanov et al. (2010) method drawback. As such, it is sensitive to specular reflections and model occlusion.

## Experiments

The proposed 2View RSfM method has been evaluated on both synthetic and real data. Synthetic evaluation considers general underwater scenario where the scene is generally situated at a distance of 2 to 4 meters to the camera. For real experiments, we consider the scenario of a consumer action camera imaging a scene situated at a close distance of approximately 0.5 meters. We also highlight a particular application for 2View RSfM and show results for fetoscopy. In such context, providing the surgeon with 3D reconstruction (as well as endoscope displacement) could facilitate both diagnosis and intervention within highly sensitive cavity such as uterus.

**Fig. 5:**
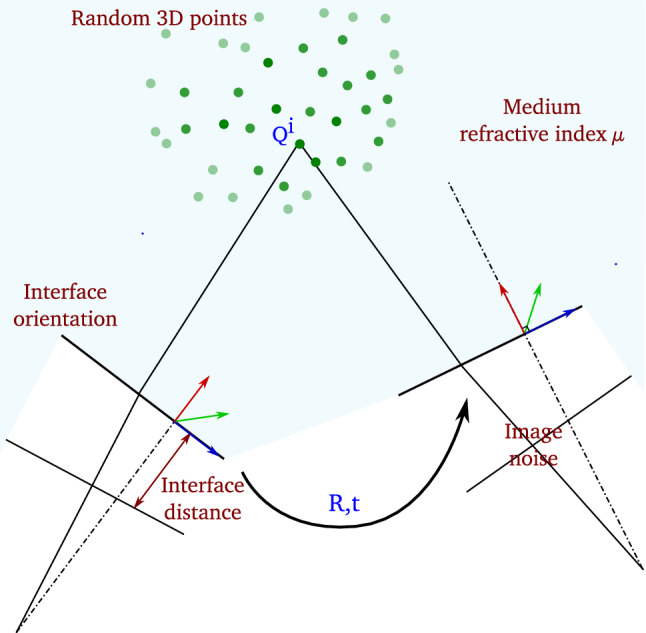
Synthetic evaluation. A set of randomly generated 3D points is projected into two views using the refractive forward projection equation (Agrawal et al. 2012). Camera motion is randomly generated. We evaluate the influence of medium refractive index, interface position and orientation as well as image noise on the robustness of our method. Parameters written in red correspond to known parameters or parameters for which we want to estimate the influence on two-view Refractive Structure-from-Motion. Parameters written in blue correspond to unknown parameters

Our code and used datasets will be published online at http://www.surgicalvision.cs.ucl.ac.uk/resources/code/.

**Fig. 6:**
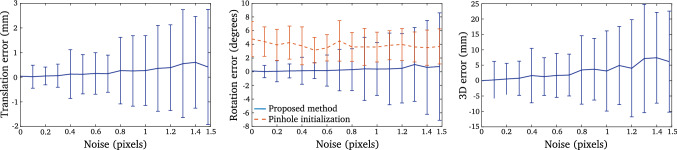
Evaluation of estimation of camera motion in the presence of noise: camera translation estimation error (left), camera rotation estimation error (center), 3D reconstruction error (right)

### Synthetic Evaluation

The synthetic dataset has been generated using the following steps (see Fig. 5). A set of 3D points situated at a distance between 2 and 4 meters from the camera are randomly generated. They are assumed to belong to a smooth 3D surface of approximate dimensions $1\times 1$ meter. For each setup, we generated 100 pairs of synthetic images considering a virtual camera moving in front of the scene. We used the refractive forward projection equation (21) derived in Agrawal et al. (2012). We considered the following internal camera parameters; focal length: 800 pixels, principal point: 640$\times$480 pixels (no distortion parameters, no skew, image size: 1280$\times$960 pixels). The refractive parameters have been randomly chosen for each pair of view. The refractive plane orientation lays in the interval $-\pi /6$ to $\pi /6$ radians (refractive plane normal rotation) and its depth in the interval 2 to 15 millimetres (mm). The camera rotation lays in the interval $-\pi /6$ to $\pi /6$ radian and its translation in the interval 0.2 to 0.5 meters (absolute distance).

A set of 40 calibration images has also been synthetically generated. We assumed a synthetic checkerboard situated at a distance of 2 to 4 meters from the camera. We generated 20 synthetic calibration images of the checkerboard in air assuming the pinhole camera model whose internal parameters have been described in the previous paragraph. We added 0.2 pixels Gaussian noise to the synthetic images of the checkerboard simulating a real calibration scenario. We also generated 20 calibration images under water (using the method described for generating the images pairs dataset) and proceed to underwater calibration considering the single viewpoint assumption (also adding a Gaussian noise of 0.2 pixels). Refractive plane calibration has not been directly carried out, as we evaluated the influence of refractive plane calibration in our experiments, it has been synthetically controlled.

**Fig. 7:**
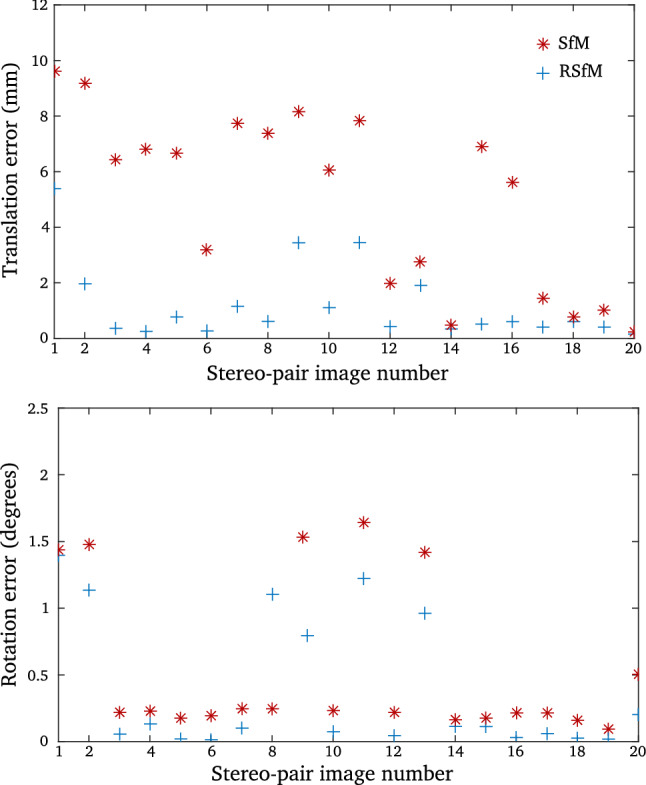
Relative pose estimation between the cameras pair of a surgical stereo-endoscope. Despite the small baseline between the two cameras, two-view Structure-from-Motion pose estimations are efficiently refined considering the refractive camera model. This is here particularly noticeable for translation estimation

We evaluated the influence of noise, medium refractive index changes and refractive plane calibration accuracy on the robustness of camera motion estimation and accuracy of 3D reconstruction (see Fig. 5). We chose to present the median and median absolute deviation errors as we noticed cases of inconsistent pose estimation using the single viewpoint assumption for noise level greater than 0.5 pixels. Considering a noise level of 1.5 pixels it appeared in $12\%$ of cases. Although the proposed method allowed us to withdraw outliers, results take into account the entire set of points.

**Fig. 8:**
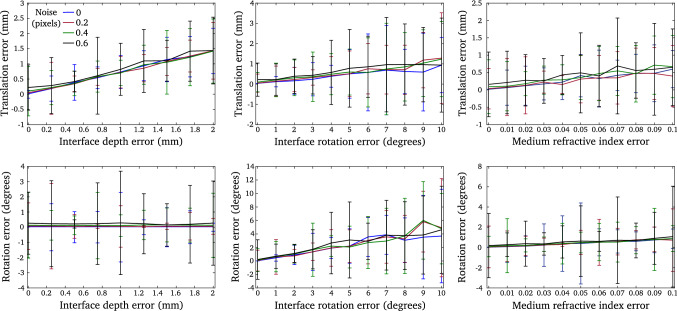
Evaluation of camera motion estimation for inaccurate calibration of the refractive interface depth (left column), the refractive interface orientation (centre column) and the refractive medium (right column). The top line presents camera translation estimation error while the second line presents camera rotation estimation error

**Robustness to Noise** we evaluated the robustness of our method to image noise. We considered a random Gaussian noise whose standard deviation lays within the range 0 to 1.5 pixels. Results are presented in Fig. 6. Despite significant noise levels, our method leads to accurate estimations of camera motions. The rotation error is less than 2 degrees despite a noise of 1.5 pixels and the translation error is approximately of 0.5 mm for the same level of noise. However, we observed a significant median absolute deviation error for noise level greater than 0.8 pixels. This was due to inconsistent initialisation leading to degenerate pose estimation. Considering a noise of 0.4 pixels we noticed 4 cases for which an inaccurate initialisation led to an alteration of camera pose estimation. For a noise between 1 and 1.5 pixels, such initialisation failures appeared in $10\%$ of evaluated cases.

Despite accurate camera motions estimations, it is important to note that above 0.6 pixels of noise, the 3D reconstruction error is greater than 2 mm. Such error can be of significance considering medical applications. This is however related to the scale of the scene as well as the setup considered in our synthetic evaluation. The evaluation of the accuracy of 3D reconstruction in the context of fluid-immersed endoscopy is highlighted in the next section.

**Robustness to inaccurate interface calibration** we first highlight the influence of inaccurate interface depth calibration on relative pose estimation (see Fig. 8, left column). The interface depth error is evaluated in the interval 0 to 2 mm. We observed that an interface depth error greater than 1 mm has a significant impact on translation estimation. The translation error is greater than 1 mm for an interface depth calibration error greater than 1.4 mm. Such errors imply inaccurate 3D reconstructions and measurements as it has been previously outlined (see Fig. 6). Interface depth errors have a minimal impact on camera rotation estimations. Moreover, we observed that noise level lower than 0.6 pixels does not significantly impact on results and behaviour of our method.

We then highlight the influence of inaccurate interface orientation calibration (see Fig. 8, central column). Refractive interface orientation accuracy has a strong influence on camera pose estimation. The translation error is greater than 0.5 mm when the interface rotation error is greater than 5 degrees. The error of camera rotation estimation is approximately 2 degrees and 4 degrees for an interface rotation error greater than 9 degrees.

**Robustness to medium refractive index changes** this evaluation is particularly relevant in the context of fluid-immersed endoscopy. For instance, most computer vision approaches for medical imaging assume organic fluid refractive index is similar to the refractive index of water while the influence of such assumption has not been quantitatively evaluated. It is particularly important for procedure such as fetoscopy which consists in inserting an endoscope (namely fetoscope) within the uterine cavity during pregnancy in order to allow access to the fetus, the placenta and the amniotic cavity. The refractive index of amniotic fluid can evolve according to gestation time despite it is mainly composed of saline fluid (Steigman et al. 2010). However, estimating the refractive index of amniotic fluid during the procedure is complex. We considered the medium refractive index error lays between 0 and 0.1. We noticed that, even for important changes of fluid refractive index, the translation estimation error is less than 0.5 mm and the rotation error less than 1 degree. These results remain valid provided that the image noise level is lower than 0.6 pixels. We therefore observed that approximating organic fluid refractive index by water refractive index remains a fair assumption despite significant changes of medium properties.

### Real Data Evaluation

#### Camera Pose Estimation Evaluation

For evaluating relative camera pose estimation, we carried out real experiments considering a surgical stereo-endoscope. We first calibrate the stereo-endoscope using a a set of 20 calibration images acquired outside water. We used a planar checkerboard and estimated both internal camera parameters and rigid pose between the cameras pair (Zhang 2000). We then followed the same procedure to calibrate the stereo-endoscope under water considering as such the adapted pinhole camera model (Nikitichev et al. 2017). We used the same set of underwater images to calibrate the orientation and distance of the flat refractive plane considering the flat refractive camera model (Agrawal et al. 2012). We finally collected a set of 20 additional images under water and compared relative camera pose estimation between the two images of the stereo pair using 2View SfM and 2View RSfM. We considered as the ground truth the rigid pose estimated during the first calibration step realised outside water. The translation between the two cameras of the stereo pair is 5.8 mm and the rotation is 3.5 degrees along the Y axis and less than a degree along the X and Z axis.

**Fig. 9:**
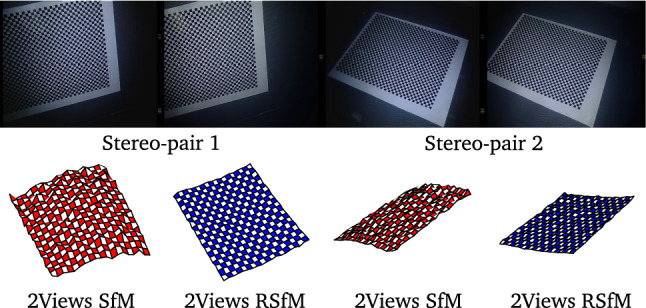
Underwater 3D reconstruction of a planar checkerboard pattern using the two views of a surgical stereo endoscope. The proposed two-view Refractive Structure-from-Motion significantly improves the initial 3D shape estimate provided by two-view Structure-from-Motion

Results are reported in Fig.e 7. We observed a mean rotation error of 0.54 degrees for 2View SfM with a variance of 0.58 degrees. Using 2View RSfM slightly improved rotation estimation, we reported a mean rotation error of 0.35 degrees and a variance of 0.51 degrees. We noticed a significant improvement of translation estimation which strengthens the results obtained for the synthetic experiments. The mean translation error for 2View SfM is 5.24 mm with a variance of 3.21 mm. The mean translation error for 2View RSfM is 1.21 mm with a variance of 1.39 mm. The translational error observed for 2View SfM can be explained by the small baseline between the cameras. However, the initial relative pose estimation provided by 2View SfM can be efficiently refined using 2View RSfM. As can be seen in Fig. 9, it has a noticeable impact on 3D shape estimation.

#### 3D Shape Estimation Evaluation

**Fig. 10:**
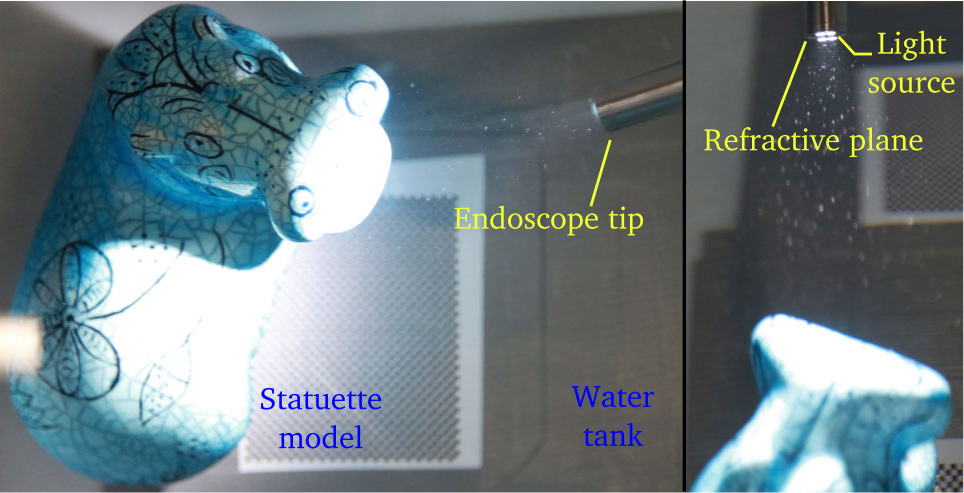
Setup used to acquire the statuette and toy dataset. The statuette dataset was realised using a Go pro hero 3 camera embedded within its dedicated watertight casing. The toy dataset was realised using a Storz® Hopkins 26008 BA fetoscope

We evaluated our approach on three datasets. For each pair of views in our datasets, we used the same feature points for both 2View SfM and 2View RSfM. Outliers have been withdrawn thanks to the method described in section 5.2. The first dataset consists of 10 images of a statuette immersed within a tank filled with water (see Fig. 10). We used a Gopro® hero 3 camera embedded within its dedicated water tight casing. The size of the statuette is approximately 150 mm width and 140 mm height and the camera was situated at a distance of approximately 400 to 500 mm. The ground truth 3D object model was obtained using an Artec® Spider 3D scanner. The second dataset consists of 15 images of a toy immersed under identical conditions. Images were acquired using a Storz® Hopkins 26008 BA fetoscope. This endoscope model relies on a rod lens optical system. Therefore, it allowed us to validate that the single refractive plane assumption approximates well a complex optical system. The size of the toy is approximately 18 mm width and 15 mm height (figurine head). The distance from the endoscope distal end to the object was approximately 20 to 50 mm. Similarly to the statuette dataset, the ground truth 3D object model was obtained using an Artec® Spider 3D scanner. The third dataset consists of 10 images of a term human placenta collected following a caesarean section delivery and immersed under water (a written informed consent was obtained at University College London Hospital (UCLH) after the Joint UCL/UCLH Committees on the Ethics of Human Research approved the study (14/LO/0863)). Images were acquired using the fetoscopic equipment previously mentioned (distance between 50 to 80 mm). Our goal was to evaluate the robustness of our approach towards 2View SfM in real fetoscopic conditions. Fetoscopy is performed during pregnancy. The placenta positions itself at either the top or side of the uterus within the amniotic sac. At the early stage of pregnancy, the amniotic sac is filled with amniotic fluid which is progressively replaced by fetal urine. As such, the placenta is always immersed within fluid during pregnancy but the nature of this fluid evolves. Unfortunately, it was impossible to obtain a reliable ground truth 3D model of the placenta. Indeed, once the placenta is immersed within water, refractive distortion prohibits the use of 3D laser scanners. Furthermore, water penetrates within the organ significantly deforming it which invalidates any 3D model computed outside water.

**Fig. 11:**
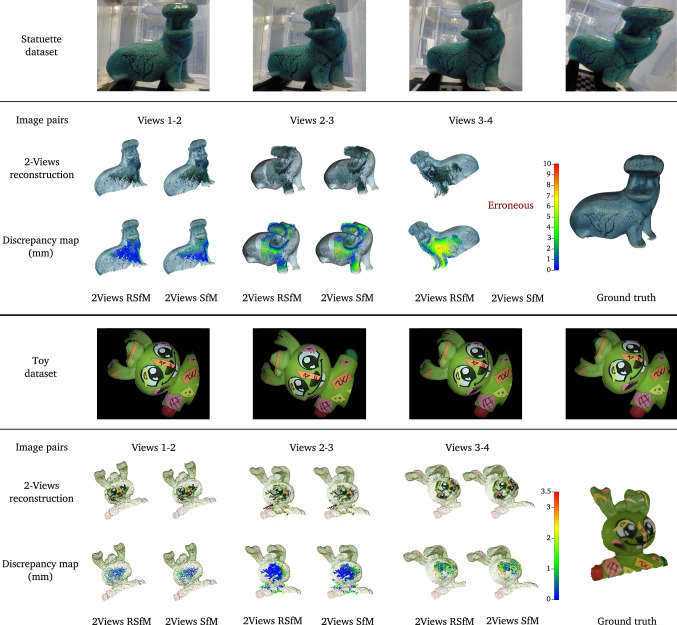
Representative sample of the statuette and toy dataset results. The top row presents three consecutive images pairs as well as the ground truth 3D reconstruction obtained thanks to a high definition Artec® Spider 3D scanner. For each of the image pair we present the results obtained with the proposed two-view Refractive Structure-from-Motion method as well as two-view Structure-from-Motion method. The second row presents 3D reconstruction results overlay on the ground truth mesh. The last row presents the discrepancy map obtained after aligning 3D reconstruction results with the ground truth mesh. Results demonstrate a sensitive improvement of 3D reconstruction accuracy using two-view Refractive Structure-from-Motion. It is worth to note for the statuette dataset that, for two cases, two-view Structure-from-Motion did not allow us to obtain reliable 3D shape reconstruction

**Fig. 12:**
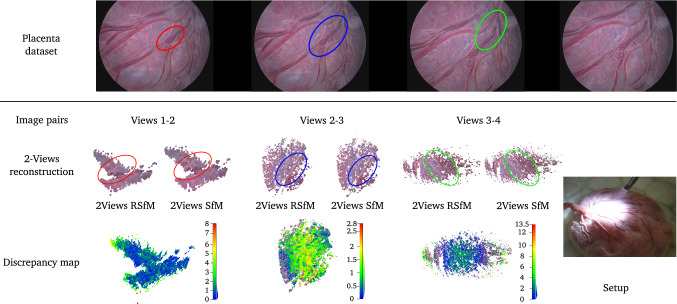
Underwater 3D reconstruction of a placenta. The top row presents three consecutive images pairs of the placenta dataset. It has been obtain by imaging a real placenta, immersed in a tank filled with water, thanks to a fetoscope. The visual appearance of the 3D reconstruction obtained thanks to the proposed two-view Refractive Structure-from-Motion (second raw) and the two-view Structure-from-Motion are similar. However it can be observed a sensitive difference while comparing these reconstructions. It is more particularly the case in the vicinity of the veins visible at the surface of the placenta (last row)

**Statuette dataset** Figure 11 depicts a representative sample of the 3D reconstruction results obtained with both 2View SfM (assuming underwater camera parameters) and our 2View RSfM method. More particularly, we present the discrepancy map between the ground truth reconstruction and these reconstructions. We observed a mean error of 5.3 mm and a standard deviation of 3.6 mm for 2View SfM while we obtained an error of 4.6 mm and a standard deviation of 3.2 mm for 2View RSfM. Beyond the accuracy improvement we noticed two particular cases where 2View SfM did not allow us to obtain reliable 3D reconstructions. Initial camera pose estimates were too erroneous in those cases to allow us to infer acceptable 3D shape of the statuette. We noticed that it corresponded to cases for which the object depth range was particularly large (side view of the statuette) or cases for which the object was located at a close distance from the camera. We nevertheless observed in such situations that our algorithm allowed us to correct for these erroneous initialisation despite a loss in accuracy (see Fig. 11).

**Toy dataset** results related to this dataset are depicted in Figure 11. We observed similar accuracy using 2View SfM and 2View RSfM with a mean error of respectively 0.3 and 0.2 mm and a standard deviation of 0.4 and 0.5 mm. It can nevertheless be highlighted a slight improvement in uniformity of shape reconstruction which can be observed by looking at the distribution of the errors. Moreover, it is worth to note that our method allowed us to efficiently remove outliers which is essential in underwater imaging.

**Fetoscopy dataset** we aimed at reconstructing the surface of a human placenta immersed under water reproducing a real fetoscopic surgery scenario. Results are presented in Fig. 12. Quasi-dense 3D reconstruction in such case remains difficult due to the lack of features at the surface of the organ. We have however been able to obtain partial reconstruction for both 2View SfM and 2View RSfM thanks to the effectiveness of the proposed method for removing outliers. While 3D shape estimation appears visually similar for both 2View SfM and 2View RSfM, we observe significant disparities in the vicinity of veins and arteries. The proposed 2View RSfM method highlights more noticeably the slight changes at the surface of the placenta which is of particular importance in fetoscopy. We furthermore noticed that it was particularly significant when the viewing angle of the camera was important (side view of the placenta) which is consistent with the results obtained with the statuette dataset.

## Conclusion

We have proposed a new formulation for the two-view geometry of images in underwater environments relying on the refractive epipolar geometry. We derived a novel rF constraint which forms the basis of a robust method for accurate and quasi-dense stereo reconstruction under water. More particularly, we showed that relative camera pose estimation based on the rF constraint outperforms state of the art techniques considered in underwater imaging. Numerical validation on synthetic data demonstrates the robustness of our approach against varying levels of additive noise while state of the art methods are particularly noise sensitive. This is an important improvement in underwater imaging context. We validated our method on real images by performing laboratory experiments and demonstrated that it improves underwater 2View SfM on both 3D shape and camera pose estimation. We demonstrated a potential application of 2View RSfM for endoscopic surgery. We successfully applied our method to fetoscopy and more particularly the 3D reconstruction of a placenta. Beyond this evaluation, the practical value of our work lies in various applications of underwater imaging where one can assume a single, thin refractive interface that separates the camera and the external environment.

Possible extensions of our work include implementing it within a more comprehensive pipeline for multiple-view reconstruction by extending our two view formulation for 2View RSfM. It would also be of interest to apply the constraint to develop a refractive Simultaneous Localisation and Mapping (SLAM) framework and potentially consider in more depth the relationship between the light source and the camera, which dictates the position of reflections within the scene’s illumination. Theoretically our current assumption of a single refractive interface may also be extended to handle thick interfaces that are used for deep underwater reconstruction. Our method could then integrate motion information generally provided by underwater robotic system as an additional constraint.

## Figures and Tables

**Fig. 13:**
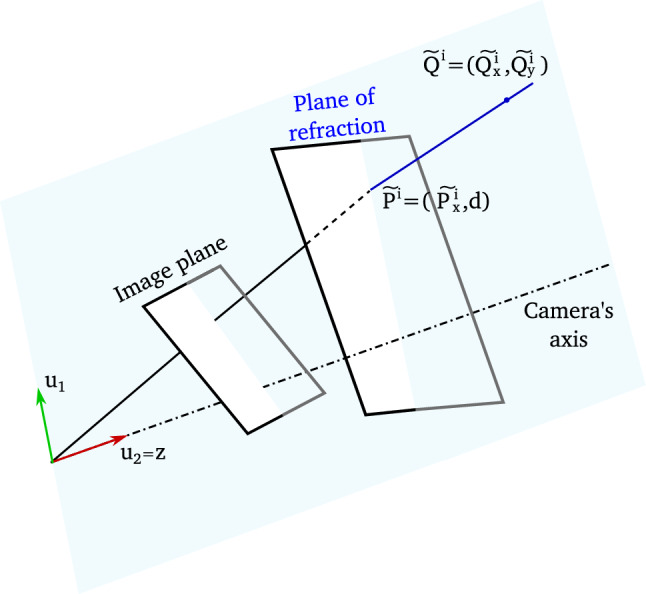
The refractive forward projection equation can be derived from the co-planarity constraint ensuring each light path lies on the plane of refraction (Agrawal et al. 2012)

## Appendix A: Refractive Forward Projection Equation

Two constraints can be directly derived from the flat refractive camera model (Agrawal et al. 2012). They are illustrated in (Fig. 13) and recalled below in order to introduce the forward refractive projection equation.

The Flat Refraction Constraint (FRC) enforces the incident light ray $\mathbf {L}_j^i$ (i.e. travelling within the external medium) to be parallel to the line joining the point $\mathsf {R}_j\mathbf {Q}^{i}+\mathbf {t}_j$ and the incidence point $\mathbf {P}_j^i$. The rotation matrix $\mathsf {R}_j$ and the translation vector $\mathbf {t}_j$ define the pose of the camera in view *j*.

12 $$\begin{aligned} (\mathsf {R}_j\mathbf {Q}^{i}+\mathbf {t}_j - \mathbf {P}_j^i) \times {\mathbf {L}_{(1,2,3)}}^{i}_{j} = 0 \end{aligned}$$

The Co-planarity Constraint (CC) enforces each light ray to lie on the plane of refraction (defined by the camera axis and an incident light ray) and the refracted ray to also intersect the camera axis. This can be mathematically described by:

13 $$\begin{aligned} (\mathsf {R}_j\mathbf {Q}^{i}+\mathbf {t}_j)^\top (\mathbf {n} \times \mathbf {P}^{i}_j) = 0 \end{aligned}$$

The refractive forward projection equation has originally been defined in Glaeser and Schröcker (2000) but it can also be efficiently derived from the CC (Agrawal et al. 2012). It allows us to compute the coordinates of the point of incidence $\mathbf {P}^{i}_j$ corresponding to the 3D point $\mathbf {Q}^{i}$. It is derived considering the 2D coordinate frame $(u_1,u_2)$ of the corresponding plane of refraction, where the axis $u_2$ aligns with the camera’s optical axis (i.e. $u_2=n=Z$) and the axis $u_1 = u_2 \times (u_2 \times \mathbf {Q}^{i})$. The point $\tilde{\mathbf {Q}}^{i}=(\tilde{\mathbf {{Q}}_{x}}^{i},\tilde{\mathbf {{Q}}_{y}}^{i})$ expresses the 3D point $\mathbf {Q}^{i}$ in this coordinate frame. Similarly, $\tilde{\mathbf {P}}^{i}_j=(\tilde{\mathbf {{P}}_{x}}^{i}_j,\tilde{\mathbf {{P}}_{y}}^{i}_j)=(\tilde{\mathbf {{P}}_{x}}^{i}_j,d)$ corresponds to the point $\mathbf {P}^{i}_j$. The refracted light ray can then be expressed by $\tilde{\mathbf {{P}}_{x}}^{i}_j u_2+du_1$. By deriving the CC in the coordinate frame of the plane of refraction, and considering a single and thin refractive interface, the refractive forward projection equation can be formulated by the following 4th degree polynomial equation in the unknown $\tilde{\mathbf {{P}}_{x}}^i_j$ (Agrawal et al. 2012):

14 $$\begin{aligned} (\tilde{\mathbf {{Q}}_{x}}^{i} - \tilde{\mathbf {{P}}_{x}}^{i}_{j})^2 (d^2 \mu ^2 + \mu ^2 {\tilde{\mathbf {P}_{x}}{^i_j}}^2) - (d\tilde{\mathbf {P}_{x}}{^i_j} - \tilde{\mathbf {Q}_{y}}^{i} \tilde{\mathbf {{P}}_{x}}{^i_j} )^2 = 0 \end{aligned}$$

where $\mu$ corresponds to the refractive index of the external medium (e.g. water refractive index $\approx$ 1.33).

## Appendix B: Single-view Refractive Geometry

For the sake of clarity, we here recall the refractive single-view geometry introduced in Chari and Sturm (2009).

An incidence point $\mathbf {P}^{i}_{1}$ lying on the refractive plane is expressed by:

15 $$\begin{aligned} \mathbf {P}^{i}_{1} = \left( {-d\frac{{p_x}_{1}^{i}}{{p_z}_{1}^{i}}} \ \ {-d\frac{{p_y}_{1}^{i}}{{p_z}_{1}^{i}}} \ \ 0 \ \ 1\right) ^\top \end{aligned}$$

reminding that $\mathcal {P}^{i}_{j} = ({p_x}_{j}^{i}\ {p_y}_{j}^{i}\ {p_z}_{j}^{i})^\top$ corresponds to the refracted light ray associated to the point $\mathbf {p}^{i}_j$ and *d* is the distance from the camera optical centre to the refractive plane.

Snell’s law (Hecht 1998) allows us to derive the direction vector of the corresponding incident light ray:

16 $$\begin{aligned} \mathcal {L}^i_1 = (\lambda {p_x}_{1}^{i} \ \ \lambda {p_y}_{1}^{i} \ \ \sqrt{1-\lambda ^2+\lambda ^2 {{p_z}_{1}^{i}}^2})^\top \end{aligned}$$

where $\lambda =\frac{\mu _1}{\mu _2}$, $\mu _1$ is the refractive index within the camera housing (generally equal to 1) and $\mu _2$ is the refractive index of the refractive medium. We furthermore define ${\gamma _z}^i_j~=~\sqrt{1-\lambda ^2+\lambda ^2 {{p_z}_{j}^{i}}^2}$.

Using Plücker coordinates, the incident light ray can therefore be formulated as:

17 $$\begin{aligned} \mathbf {L}^i_1 = (\lambda {p_x}_{1}^{i} \ \ \lambda {p_y}_{1}^{i} \ \ {\gamma _z}^i_1 \ \ -d\frac{{p_y}_{1}^{i}}{{p_z}_{1}^{i}}{\gamma _z}^i_1 \ \ d\frac{{p_x}_{1}^{i}}{{p_z}_{1}^{i}}{\gamma _z}^i_1 \ \ 0)^\top \end{aligned}$$

The Plücker constraint on lines intersection directly derives from the definition of Plücker coordinates (Sturm and Barreto 2008). It allows us to express the intersection of the incident ray $\mathbf {L}^i_1$ with a 3D ray $\mathbf {L'}$ (i.e. a 3D ray or a 3D line $\mathcal {L'}$ intersecting the incident ray $\mathcal {L}_i^1$):

18 $$\begin{aligned} {\mathbf {L}^i_1}^\top \mathsf {W} \mathbf {L'} = 0 \end{aligned}$$

where $$\mathsf {W} = \left( \begin{array}{cc} 0 &{} \mathsf {I} \\ \mathsf {I} &{} 0 \end{array} \right) $$.

The previous relationship can be rewritten in the following form:

19 $$\begin{aligned} \lambda {\mathbf {L'}^\top _{(4,5,3)}}^{i}_{1} t_a {\mathcal {P}}^{i}_{1} + \frac{{\gamma _z}^i_1}{{p_z}_{1}^{i}} {\mathbf {L'}^\top _{(6,1,2)}}^{i}_{1} t_b {\mathcal {P}}^{i}_{1} = 0 \end{aligned}$$

where $$t_a=\left( \begin{array}{ccc} 1 &{} 0 &{} 0 \\ 0 &{} 1 &{} 0 \\ 0 &{} 0 &{} 0 \end{array} \right) $$ and $$t_b=\left( \begin{array}{ccc} 0 &{} 0 &{} 1 \\ 0 &{} -d &{} 0 \\ d &{} 0 &{} 0 \end{array} \right) $$.

Removing root square in ${\gamma _z}^i_1$ allows to reformulate equation (19) as follow (Chari and Sturm 2009).

20 $$\begin{aligned} \left( \lambda ^2 (-\hat{\mathsf {A}} + \hat{\mathsf {B}}) \ \ (1-\lambda ^2) \hat{\mathsf {B}}\right) \underbrace{\left( \begin{array}{cc} \mathsf {D}_{t} &{} 0 \\ 0 &{} \mathsf {D}_t \end{array} \right) }_{\mathsf {D'}} ({\widehat{\mathcal {P}}^{i}_{1}} \ \ \frac{{\widehat{\mathcal {P}}^{i}_{1}}}{{{p_z}_{1}^{i}}^2})^\top = 0 \end{aligned}$$

where $$ \mathsf {A} = \lambda {\mathbf {L'}^\top _{(4,5,3)}}^{i}_{1} \mathsf {t}_a$$ and $$\mathsf {B} = {\mathbf {L'}^\top _{(6,1,2)}}^{i}_{1} {\mathsf {t}_b}$$.

The refractive projection equation can then be derived in its general form by expending equation (20). Unlike the formulation proposed in Agrawal et al. (2012) (equation (14)), it does not directly depend on the planes of refraction.

21 $$\begin{aligned} \left( {\widehat{\mathbf {L'}}}{^\top _{(6,1,2)}}{^i_1} \ \ {\widehat{\mathbf {L'}}}{^\top _{(4,5,3)}}{^i_1} \right) \mathsf {rP}^\top \begin{pmatrix} \frac{\widehat{\mathcal {P}}^{i}_{1}}{{{p_z}{^i_1}}^2} \\ {\widehat{\mathcal {P}}^{i}_{1}} \end{pmatrix}=0 \end{aligned}$$

The refractive projection matrix $\mathsf {rP}$ is therefore defined in Chari and Sturm 2009 as:

22 $$\begin{aligned} \begin{aligned} \mathsf {rP}&= \mathsf {D'}^\top \underbrace{ \left( \begin{array}{cc} (1-\lambda ^2) \mathsf {D}_t^{-1}\mathsf {S}_t\mathsf {t}_b^\top \otimes \mathsf {t}_b^\top \mathsf {S}_t^\top &{} 0 \\ \\ \lambda ^2 \mathsf {D}_t^{-1}\mathsf {S}_t\mathsf {t}_b^\top \otimes \mathsf {t}_b^\top \mathsf {S}_t^\top &{} -\lambda ^2 \mathsf {D}_t^{-1}\mathsf {S}_t\mathsf {t}_a^\top \otimes \mathsf {t}_a^\top \mathsf {S}_t^\top \end{array} \right) }_{\mathsf {M}} \end{aligned} \end{aligned}$$

The refractive projection matrix is of size $12\times 12$. It only depends on the medium refractive index on both sides of the refractive interface as well as on the distance between the optical centre of the camera and the refractive plane.
